# Minimally Invasive Lumbar Pedicle Screw Fixation Using Cortical Bone Trajectory – A Prospective Cohort Study on Postoperative Pain Outcomes

**DOI:** 10.7759/cureus.714

**Published:** 2016-07-26

**Authors:** Yi-Ren Chen, Sayantan Deb, Lan Pham, Harminder Singh

**Affiliations:** 1 Department of Neurosurgery, Stanford University Medical Center; 2 Medical School, Stanford University School of Medicine

**Keywords:** cortical screws, outcomes, prospective cohort study, postoperative pain

## Abstract

Objective: Our study aims to evaluate the clinical outcomes of cortical screws in regards to postoperative pain.

Background: Pedicle screw fixation is the current mainstay technique for posterior spinal fusion. Over the past decade, a new technique called cortical screw fixation has been developed, which allows for medialized screw placement through stronger cortical bone. There have been several studies that showed either biomechanical equivalence or superiority of cortical screws. However, there is currently only a single study in the literature looking at clinical outcomes of cortical screw fixation in patients who have had no prior spine surgery.

Methods: We prospectively looked at the senior author’s patients who underwent cortical versus pedicle lumbar screw fixation surgeries between 2013 and 2015 for lumbar degenerative disease. Eighteen patients underwent cortical screw fixation, and 15 patients underwent traditional pedicle screw fixation. We looked at immediate postoperative pain, changes in short-term pain (six to 12 weeks post-surgery), and changes in long-term pain (six to eight months). All pain outcomes were measured using a visual analog scale ranging from 1 to 10. Mann-Whitney or Kruskal-Wallis tests were used to measure continuous data, and the Fisher Exact test was used to measure categorical data as appropriate.

Results: Our results showed that the cortical screw cohort showed a trend towards having less peak postoperative pain (p = 0.09). The average postoperative pain was similar between the two cohorts (p = 0.93). There was also no difference in pain six to 12 weeks after surgery (p = 0.8). However, at six to eight months, the cortical screw cohort had worse pain compared to the pedicle screw cohort (p = 0.02).

Conclusions: The cortical screw patients showed a trend towards less peak pain in the short-term (one to three days post-surgery) and more pain in the long-term (six to eight months post-surgery) compared to pedicle screw patients. Both cohorts had a statistically significant reduction in pain levels compared to preoperative pain. More studies are needed to further evaluate postoperative pain, long-term functional outcomes, and fusion rates in patients who undergo cortical screw fixation.

## Introduction

**Figure 1:**
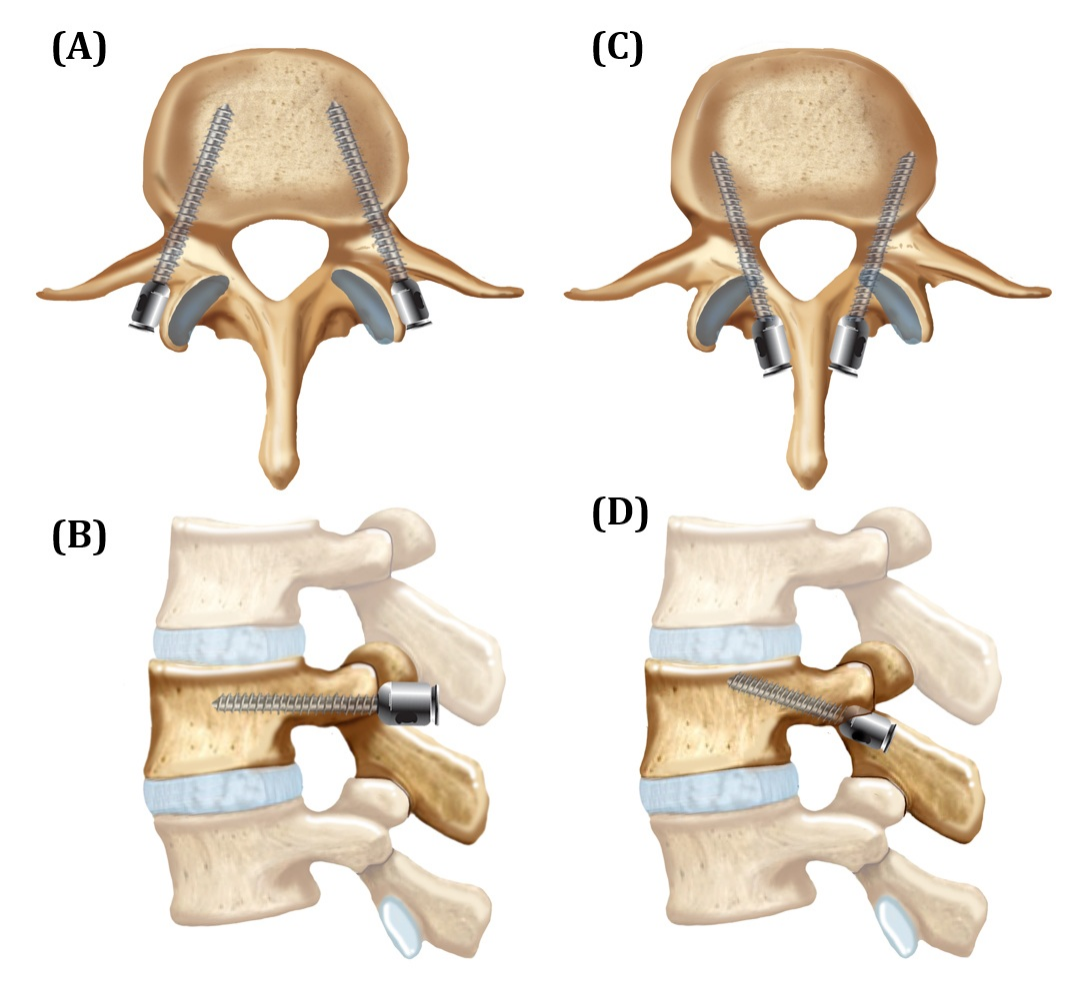
Cortical Versus Pedicle Screw Fixation Trajectories (A and B) Traditional pedicle screw trajectories in (A) axial and (B) sagittal views. (C and D) Cortical screw trajectories in (C) axial and (D) sagittal views.

Every year, over 120,000 lumbar fusions are performed nationwide for degenerative and traumatic spine conditions [1]. The fusions are mostly done with pedicle screw fixation, the current standard technique for accomplishing posterior spinal fusion, due to its reliable fusion rates and construct stability. However, the technique is invasive and requires significant lateral spinal dissection in order to properly place the screws, resulting in large incisions and long operative times. In keeping with the push to perform more minimally invasive spine surgery, a new technique of spinal instrumentation has been developed whereby screws are placed through a starting point at the junction of the superior articular process and pars. This technique is called cortical screw fixation (Figure 1).

Over the past decade, there have been several studies that showed either biomechanical equivalence or superiority of cortical screws compared to pedicle screws [2-11]. One study by Baluch, et al. looked at 17 vertebral levels that underwent quantitative computed tomography (CT) [2]. On one side, cortical screws were placed, and on the other, traditional pedicle screws were placed. Cortical screws demonstrated significantly improved resistance to toggle testing, requiring 184 cycles to reach 2 mm of displacement compared to 102 cycles for the traditional pedicle screws (p = 0.002).

**Figure 2:**
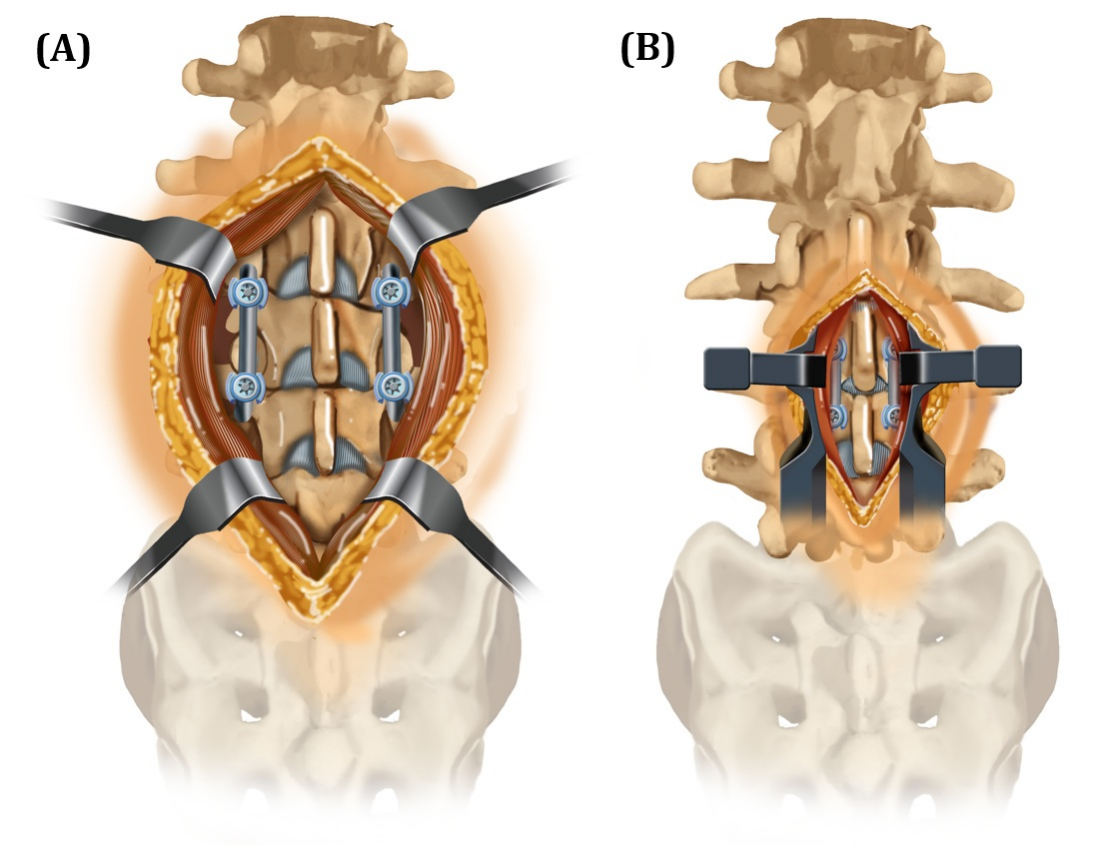
Soft Tissue Exposure Required for One-Level Lumbar Fixation (A) Traditional pedicle fixation. (B) Cortical screw fixation.

Due to the promising biochemical studies and the minimally invasive nature of cortical screws, some surgeons are starting to utilize the technique in lumbar fusions. However, there is currently only one study in the literature looking at the clinical efficacy or outcomes of cortical screws in non-redo patients [12]. In that prospective randomized non-inferiority trial, Lee, et al. showed that cortical screw fixation in posterior lumbar interbody fusion (PLIF) provides similar clinical and radiological outcomes compared to pedicle screw fixation. Most past studies have reported on biomechanical strength and not on clinical outcomes [2-10]. In this study, we focus on postoperative and long-term pain in patients who underwent cortical versus pedicle screw fixation in the lumbar spine. Our goal was to evaluate the hypothesis that cortical screw patients should have less postoperative pain due to the smaller incision, less dissection needed to find entry points, and more intraoperative preservation of muscle attachments (Figure 2). Low back pain is often related to muscular stabilization of the “neutral zone” in the back, and the lumbar multifidus muscles are important stabilizers of this neutral zone. Studies have shown that dysfunction of these muscles, such as after surgery, is associated with increased pain [13].  

## Materials and methods

### Cohort selection

We prospectively enrolled patients with lumbar degenerative disease and instability who needed lumbar fusion from 2013 to 2015 at the Santa Clara Valley Medical Center (SCVMC) in San Jose, CA. SCVMC is a level one trauma center and the county hospital wing of Stanford University Medical Center. IRB approval for the prospective data registry was obtained through SCVMC (approval #15-026ER). Informed patient consent was obtained at the time of treatment. All patients underwent pedicle or cortical screw placement by the senior author. Patients who underwent surgery because of trauma or infection, such as osteomyelitis, were excluded. In total, 18 patients underwent cortical screw fixation, and 15 underwent pedicle screw fixation. Most of the patients in the lumbar pedicle screw fixation group had their spinal fixation surgeries *before* the institution of lumbar cortical trajectory screws at SCVMC. The only patients who had pedicle screw fixation done *after* the institution of cortical trajectory screws were the ones with small pedicles (< 7 mm diameter) on preoperative CT scans.

All patients received the same pain regimen postoperatively, with a morphine patient-controlled analgesia pump for 24 hours, followed by long-acting OxyContin and IV morphine and/or Percocet thereafter during the hospital stay. Patients were discharged home on oral Percocet.

### Surgical technique

Traditional pedicle screws were placed using the technique described by Weinstein, et al. [14]. A standard skin incision was made and lateral muscle dissection was performed to expose the transverse processes. An awl was used to breach the cortex at the lateral facet surface, and a pedicle finder was used to extend the trajectory. A tap was then used and screws placed. Overall, a total of 23 levels were fused in the pedicle group. In addition, arthrodesis was also performed in the lateral gutters over the transverse processes. No interbody grafts were used.

The cortical screw starting point is at the lateral aspect of the pars interarticularis and, therefore, requires significantly less lateral muscle dissection. The angulation is medial to lateral, rather than lateral to medial as in the traditional pedicle screw technique. Screws are inserted approximately 10 degrees laterally in the axial plane and 25 degrees cranially in the sagittal plane, although actual angulations are determined by intraoperative fluoroscopy (Figure 1) [4]. Pedicles, 7 mm in diameter, were used as the minimum cut-off in order to safely perform cortical screw fixation without lateral vertebral body breach. A total of 26 levels were fused in the cortical group. In addition, arthrodesis was also performed over the facet joints with a high-speed drill.

### Outcome measurements

We looked at peak pain and average immediate postoperative pain from 24 to 72 hours after surgery. We also looked at changes in short-term pain (six to 12 weeks post-surgery) and changes in long-term pain (six to eight months), when compared to preoperative pain in the two cohorts. All pain outcomes were measured using a visual analog scale ranging from 1 to 10.

### Statistical analysis

Mann-Whitney or Kruskal-Wallis tests were used to analyze the difference in pain outcomes between the two groups and over time, respectively. Categorical data was analyzed using Fisher’s exact test. A p - value of less than 0.05 was considered statistically significant. GraphPad Prism v 6.0 was used to conduct all statistical analyses.

## Results

A total of 36 patients were included in the study. Three were excluded because of trauma and infection being the indications for fusion, with a total of 33 patients remaining. Eighteen patients underwent cortical screw fixation and 15 underwent pedicle screw fixation. Overall, the patient characteristics in the cortical versus the pedicle screw groups were similar (Table 1).

**Table 1:** Patient Characteristics ** indicates statistically significant

	Cortical Screw (n = 18)	Pedicle Screw (n = 15)	P-value
Age (mean ± SE)	53.39 ± 1.97	59.2 ± 3.12	0.12
Female (%)	38.9	86.7	0.01**
Ever Smoked (%)	50	46.7	1.00
Preoperative Pain (mean ± SE)	7.61 ± 0.36	7.43 ± 0.44	0.79

Age, preoperative pain, smoking status, and comorbidities were all similar, with the exception of gender; the pedicle screw group had more females (86.7%) compared to the cortical screw group (38.9%) (p = 0.01). Degenerative disc (DD) disease was the predominant finding on MRI in both cohorts, and presenting pain symptoms were also similar (Table 2).

**Table 2:** Patient Pathology, Presenting Symptoms, and Levels Fused DD - degenerative disease; NFS - neuroforaminal stenosis; b/l - bilateral

Pathology	Presenting Pain Symptoms	Levels Fused
Cortical Screw Series		
Central and NFS: L5-S1 spondylosis with b/l pars defect	Back pain and bilateral radicular leg pain	L5-S1
DD and NFS at L4-5 and L5-S1	Back pain and bilateral radicular leg pain	L4-S1
Central stenosis L4-5, b/l NFS L5-S1	Back pain and left radicular leg pain	L4-S1
Severe central stenosis with Grade 1 anterolisthesis, b/l NFS	Back pain and bilateral radicular leg pain	L4-L5
Central stenosis, left NFS - facet hypertrophy + synovial cyst	Back pain and left radicular leg pain	L4-L5
DD and NFS at L4-5 and L5-S1	Back pain and bilateral radicular leg pain	L4-S1
Central stenosis and left NFS L5-S1	Back pain and neurogenic claudication	L5-S1
DD and right NFS	Back pain and bilateral radicular leg pain	L4-S1
DD and left NFS	Back pain and left radicular leg pain	L4-L5
DD and left NFS - L3-4 and L4-5	Back pain and left radicular leg pain	L3-S1
DD and left NFS	Back pain and left radicular leg pain	L4-L5
Central stenosis, left NFS	Back pain and left radicular leg pain	L4-L5
DD and central stenosis, L4-5 and L5-S1	Back pain and right radicular leg pain	L4-S1
Central stenosis, NFS at L3-4 and L4-5 + synovial cyst	Back pain and right radicular leg pain	L3-L5
DD and right NFS	Back pain and right radicular leg pain	L4-S1
DD and left NFS	Back pain and bilateral radicular leg pain	L4-5
Central stenosis, left NFS	Back pain and left radicular leg pain	L4-5
L5-S1 spondylosis with b/l NFS	Back pain and right radicular leg pain	L5-S1
Pedicle Screw Series		
DD and central and right NFS L5-S1	Back pain and bilateral radicular leg pain	L5-S1
Central stenosis and b/l NFS	Back pain and bilateral radicular leg pain	L3-L4
Central stenosis and left NFS	Back pain and left radicular leg pain	L4-S1
Central stenosis and b/l NFS L3-5	Back pain and bilateral radicular leg pain	L3-S1
L4-S1 central stenosis and right NFS	Low back pain and right radicular leg pain	L4-S1
DD and central stenosis	Low back pain and neurogenic claudication	L2-L3
L3-5 central stenosis and b/l NFS	Back pain and bilateral radicular leg pain	L3-L5
Central stenosis and b/l NFS, anterolisthesis L4 on L5	Back pain and left radicular leg pain	L4-L5
Central stenosis and right NFS	Low back pain and right radicular leg pain	L4-S1
Central stenosis and b/l NFS	Back pain and bilateral radicular leg pain	L4-5
DD and central stenosis	Back pain and bilateral radicular leg pain	L4-S1
L5 on S1 anterolisthesis and b/l NFS	Back pain and bilateral radicular leg pain	L5-S1
Central stenosis with b/l NFS	Back pain and Left radicular leg pain	L4-S1
Stage II anterolisthesis L3 on L4	Back pain and neurogenic claudication	L3-4
Central stenosis and anterolisthesis L4 on L5	Back pain and bilateral radicular leg pain	L4-5

**Figure 3:**
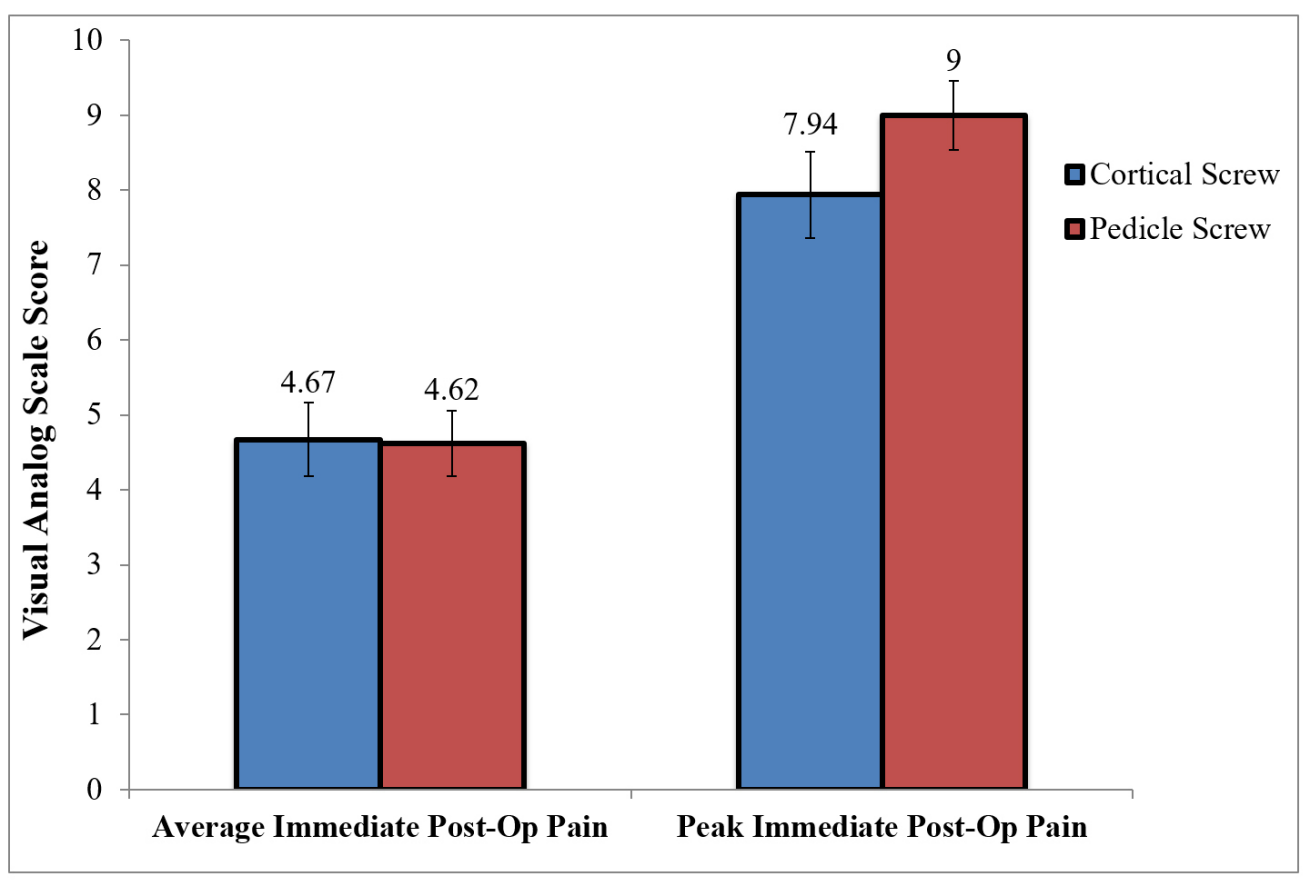
Immediate Postoperative Pain in Cortical Versus Pedicle Screw Patients

Overall, there was no difference in average (p = 0.93) or peak (p = 0.09) immediate postoperative pain between patients who underwent cortical or pedicle screw fixation, as seen in Figure 3. However, there was a trend towards cortical screw patients having less peak postoperative pain, at a pain score of 7.94 versus 9 (p = 0.09).

There was no difference in postoperative pain at the time of short-term follow-up at six to 12 weeks, with an average pain score of 4.97 in the cortical group compared to 4.93 in the pedicle group (p = 0.8) (Figure 3).

**Figure 4:**
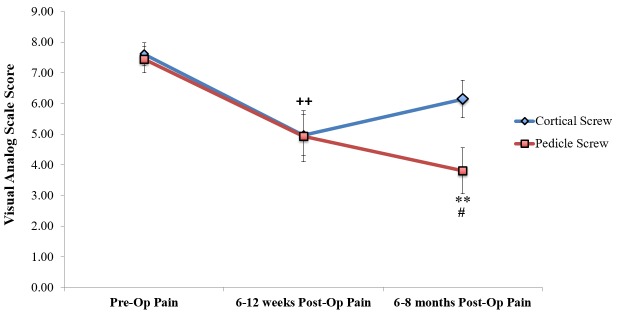
Long-Term Postoperative Pain in Cortical Versus Pedicle Screw Patients

However, the cortical screw patients did have more pain at the six to eight-month follow-up, with a pain score of 6.14 compared to 3.8 in the pedicle group (p = 0.02) (Figure 4).

Both groups had a statistically significant reduction in pain levels compared to preoperative pain, as seen in Table 3.

**Table 3:** Numerical Data and Statistical Analysis of Long-term Postoperative Pain in Cortical Versus Pedicle Screw Patients ** indicates levels of significance

	Preop	6-12 weeks	6-8 months	p-value (Kruskal-Wallis) over time within group	Multiple comparisons
Mean Pain Cortical Screw (mean ± SE)	7.61 ± 0.36	4.97 ± 0.68	6.14 ± 0.61	0.004	Pre-op vs. 6-12 weeks**
Mean Pain Pedicle Screw (mean ± SE)	7.43 ± 0.44	4.93 ± 0.84	3.80 ± 0.75	0.002	Pre-op vs. 6-8 months**
p-value Between Groups	0.79	0.80	0.02**		

The raw data for all patients in the study are shown in Tables 4-5.

**Table 4:** Cortical Screw Patient Series COPD – chronic obstructive pulmonary disease; DM – diabetes mellitus; HTN – hypertension; HLD – hyperlipidemia; OA – osteoarthritis; CKD – chronic kidney disease; GERD – gastroesophageal reflux disease; NA – Data not available

Age	Immediate Average Postop Pain	Immediate Peak Postop Pain	6-12 Weeks Postop Pain	6-8 Months Postop Pain	Ever Smoked	Significant Comorbidities
50	5.733	10	7	7	yes	Neuropathy
44	6.313	9	6	5	yes	COPD
42	3.222	7	4	NA	no	Chronic neck pain
59	5.5	10	0	5	yes	DM, HTN, HLD
59	4.958	10	0	6	yes	DM, OA, Sciatica
67	5.375	10	0	6	no	HTN, HLD
52	2.875	6	7	8	yes	HTN, HLD, DM
57	8.286	10	7	10	no	HTN
35	8.5	9	9	8	yes	Seizures
50	6.154	9	7	7	no	DM, HTN, CKD
56	4.25	8	6	7	no	Lumbago
64	2.4	9	4	5	no	DM, HTN
58	3.429	6	6	7	yes	HTN
44	4	7	5	0	yes	HTN
49	4.077	7	4	5	yes	DM, HTN
55	3.133	7	4	NA	no	GERD
59	0	0	8.5	NA	no	DM, Migraine
61	6	9	NA	NA	no	Fibromyalgia

**Table 5:** Pedicle Screw Patient Series CHF – congestive heart failure; DM – diabetes mellitus; HTN – hypertension; HLD – hyperlipidemia; OA – osteoarthritis; CKD – chronic kidney disease; CAD – coronary artery disease; RA – rheumatoid arthritis; NA – Data not available

Age	Immediate Average Post-Op Pain	Immediate Peak Post-Op Pain	6-12 Weeks Post-Op Pain	6-8 Months Post-Op Pain	Ever Smoker	Significant Comorbidities
47	7.167	9	9	7	yes	HTN, Neuropathy, Lumbago, Seizures
77	5.6	10	7	5	no	CHF
56	4.615	10	4	6	yes	HTN, HLD, OA
49	6.286	10	9	5	yes	HTN, Seizures, Sciatica
57	5.4	10	4	0	no	Epilepsy, Sciatica, OA
71	4.364	10	7	9	no	OA, DM, HTN, CKD
78	5.222	10	6	3	no	DM, HTN, HLD
63	1.7	4	NA	2	no	HTN, HLD, OA
63	4.1538	8	0	0	yes	CAD
65	4.9444	9	5	4	yes	HLD, HTN, DM
38	4.0714	9	5	0	no	Lumbar stenosis
62	5.375	10	0	0	yes	DM, HTN, HLD CHF
39	5	10	8	7	no	DM
65	0.81	7	0	4	no	RA
58	NA	NA	5	5	yes	HTN, OA

## Discussion

Multiple biomechanical studies have shown equivalence or superiority of the cortical bone trajectory compared to the standard technique for pedicle screw fixation [2-3, 5-10]. Most studies show that cortical screws traverse denser cortical bone and, thus, result in increased pullout strength and improved rigidity. This has theoretical advantages for patients with osteoporosis, failed fusion requiring reoperation, and even first-time fusions for degenerative disease and spinal instability. However, there is only one study looking at the efficacy and clinical outcomes of cortical screw fixation in previously non-instrumented patients. In their prospective randomized trial, Lee, et al. showed that cortical screw fixation with interbody fusion provides comparable pain reduction and fusion rates to that of pedicle screw fixation with interbody fusion.

Another study in the literature that is non-cadaveric is by Rodriguez, et al. [15]. The authors retrospectively reviewed five patients who underwent cortical screw fixation and posterior interbody grafting for adjacent segment lumbar disease. All cases were reoperations after prior lumbar instrumentation. The average age of the patients was 69.4, and all five patients reported improved low back pain compared with preoperative pain at 10- to 15-month follow-up. The authors concluded that cortical screw fixation was a good technique in patients requiring a reoperation because it obviates the need for previous hardware removal.

Our paper is the first prospective cohort study looking at cortical versus pedicle screw fixation in patients with lumbar degenerative disease and spinal instability, with or without an interbody fusion. None of the cases had prior instrumentation, as we sought to evaluate the pain outcomes in patients who underwent cortical screw fixation as the first-line technique.

Overall, there was no statistically significant difference in either average or peak immediate postoperative pain. However, there was a trend towards the cortical screw patients having less peak immediate postoperative pain (average pain score of 9 versus 7.94 in the pedicle group) (p = 0.09). This is consistent with the hypothesis that a smaller incision with less muscle detachment and soft-tissue dissection in cortical screws may lead to less postoperative pain. This is also consistent with the findings in the Lee, et al. study where cortical screws were associated with lower immediate postoperative pain (within one week of surgery) compared to pedicle screws [12].

Although there was no difference in pain between cortical and pedicle screw patients at the six to 12-week follow-up, pedicle screw patients appeared to have less pain at the six to eight-month follow-up (pain score of 6.14 in cortical patients versus 3.8 in pedicle patients) (p = 0.02). This is an interesting finding, as most of the biomechanical studies have shown superior pullout strength and stability in cortical screws compared to pedicle screws [6].

One explanation for these phenomena might be that, even though cortical screws are biomechanically stronger and prevent spinal micromotion and, therefore, pain generation in the short-term, long-term stability of the construct depends on the formation of a stable fusion mass. This might be happening more effectively in the pedicle screw cohort with posterolateral fusion in the lateral gutters, compared to the cortical screw cohort where arthrodesis was performed over the facet joints, but not in the lateral gutters. Although plain radiographs did not show any hardware failure in either group at the six to eight-month follow-up, long-term follow-up is needed to see if cortical screw patients have higher levels of pseudoarthrosis compared to pedicle screw patients and whether the difference in pain outcomes between the two cohorts remains divergent into the future. Notably, Lee, et al. did not find any difference in the pain scores or fusion rates between their two cohorts at one-year follow-up [12]. However, it has to be noted that they performed an interbody fusion (PLIF) in all of their patients, so their construct stability was boosted by the interbody fusion and was not solely reliant on the lateral gutter formation of a fusion mass in the long-term.

There are some limitations to our study, including the small sample size and the lack of randomization. Although patient characteristics overall were similar between the two groups, the pedicle screw group did have a higher percentage of female patients compared to the cortical group (Table 1). This may be the result of females having smaller diameter pedicles, thus, limiting the feasibility of the cortical trajectory technique. A 7-mm diameter was used as the minimum cut-off for performing cortical screw fixation in our study. Placing cortical trajectory lumbar screws in narrow pedicles is extremely challenging because of the medial to lateral course of the screw within the pedicle. The incidence of lateral vertebral breach with cortical screws seen in cadaveric specimens with narrow pedicles was found to be very high in the lab. This difference in the sex ratio between the two cohorts may confound the data, as women may have a different pain threshold compared to men due to mood, sex-role beliefs, or hormonal effects [16]. The study also only included patients from a single center with surgery performed by a single surgeon.

## Conclusions

Our paper is the first prospective cohort study comparing pain outcomes for 33 patients who underwent cortical versus pedicle screw fixation in lumbar degenerative disease, with or without interbody fusion. Overall, cortical screw fixation results in similar to improved immediate postoperative pain but showed a trend towards worsening low back pain at the six to eight-month follow-up compared to pedicle screw patients. Both cohorts had statistically significant reduction in pain levels compared to preoperative pain after surgery (cortical at six to 12 weeks and pedicle at six to eight months). Our paper is a pilot study, and more prospective randomized clinical studies are needed to further evaluate postoperative pain, long-term functional outcomes, and fusion rates in patients who undergo cortical screw fixation.
